# Presentation and treatment of uterine leiomyoma in adolescence: a systematic review

**DOI:** 10.1186/s12905-015-0162-9

**Published:** 2015-01-22

**Authors:** Rafael Mendes Moroni, Carolina Sales Vieira, Rui Alberto Ferriani, Rosana Maria dos Reis, Antonio Alberto Nogueira, Luiz Gustavo Oliveira Brito

**Affiliations:** Department of Gynecology and Obstetrics – Ribeirao Preto Medical School, University of Sao Paulo, Avenida Bandeirantes, 3900 – 8th floor – Monte Alegre, Ribeirão Preto, SP Brazil

**Keywords:** Uterine leiomyoma, Treatment, Adolescence

## Abstract

**Background:**

Uterine leiomyoma is the most common gynecological tumor in the reproductive years. However, it is extremely rare in adolescence (<1%), with few reports found in the literature. The biological behavior of such tumors in this age group is unknown, as well as the best possible treatment for this population. We aimed to analyze all available reports of uterine leiomyoma in adolescence.

**Methods:**

A systematic review was performed at PubMed/MEDLINE and EMBASE. Between 1965 and 2014, 19 reports were found on uterine leiomyoma in patients under 18 years. The following parameters were discussed: age, tumor diameter, symptoms, clinical treatments, surgical treatments, hemodynamic changes.

**Results:**

Mean age was 15.35 (14–17) years. Mean tumor diameter was 12.28 cm (3–30) and median diameter was 10 cm. Most patients presented with symptoms (87.5%), including abnormal uterine bleeding (10/18) and pelvic/abdominal pain (6/18). A pelvic mass was the most common finding. Two patients required transfusion due to anemia. One patient underwent abdominal hysterectomy, and the others underwent myomectomy. Mean follow-up was 1 year and 8 months, and only case recurred, after 6 months.

**Conclusion:**

Leiomyomas’ biologic behavior in adolescents may be different from that of older women, but their molecular characteristics still haven’t been analyzed. Optimal treatment is still not defined, but myomectomy has several advantages in this population. Leiomyomas must be remembered as an important differential diagnosis of pelvic mass in adolescents.

## Background

Uterine leiomyomas, or fibroids, are benign tumors originating from smooth muscle cells of the uterine wall. They are exceedingly common among women of reproductive age, and it’s estimated that about 60% to 80% of women may be affected at 50 years of age. The etiology of such tumors is not well understood, but it is known that they share a monoclonal origin, i.e. they are derived from a single precursor cell. The mechanism by which these lesions rise is not known, but many factors are recognized as growth promoters, with sex steroids being the most frequently studied.

Many risk factors are associated with the development of fibroids, such as nulliparity, obesity and early menarche, with a greater exposure to sex steroids, especially estrogen, as the alleged mechanism by which they exert such action [1]. Although they are very common among women in general, leiomyomas are infrequently diagnosed in children and adolescents, with few reported cases in the literature. Nevertheless, they are important differential diagnoses in adolescents with pelvic masses. The present review aims at analyzing the reported cases of fibroids in adolescents and discuss the therapeutic strategies employed in these cases.

## Methods

**Figure 1:**
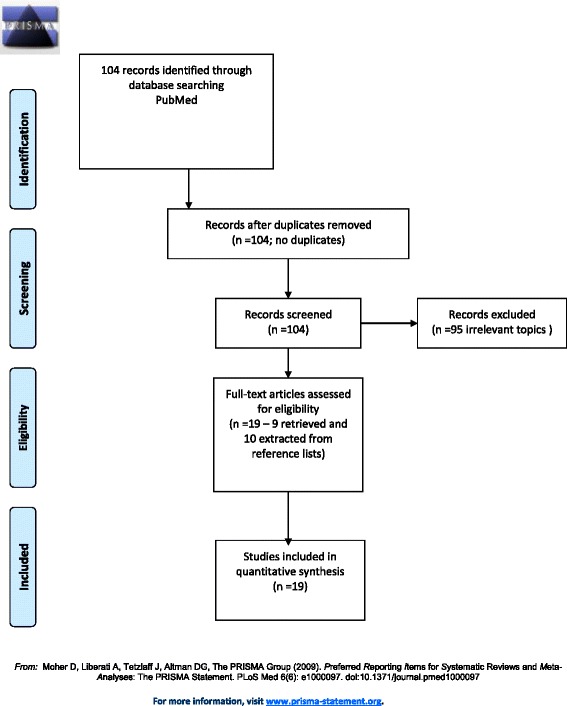
**PRISMA 2009 flow diagram.**

A search was conducted on the PubMed/MEDLINE database with the string ‘(“uterine leiomyoma” OR “uterine fibroids”) AND adolescent’ (without the external quotes), and on the EMBASE database with the string ‘(“uterine leiomyoma*” OR “uterine fibroid*”) AND adolescent*. There were 104 results found, among which 9 case reports of fibroids in adolescents were identified. Full texts of these reports were obtained and their reference lists were reviewed, leading to another 10 cases, comprising a total of 19 reports of leiomyomas in adolescents (Figure 1). Cases included were those reporting fibroids in women younger than 18 years of age.

## Results

Literature search led to 19 case reports of fibroids [2-20] among adolescents (Table 1). The first reported case was made by Wisot *et al.* in 1969, in which a 15 year old girl underwent an abdominal myomectomy due to intense vaginal bleeding and to a pelvic mass suspicious for leiomyosarcoma. There was a recurrence of the lesion six months after surgery [2].

**Table 1:** **Reported cases of uterine leiomyoma in adolescents listed in chronological order**

**Author**	**Age**	**Year**	**Clinical picture**	**Treatment**	**Tumor size (cm)**
Wisot et al. [2]	15	1969	AUB; pelvic mass	Abdominal myomectomy	Not informed
Augensen [3]	15	1981	AUB; pelvic mass; urinary retention	Abdominal myomectomy	10
De Rooy et al. [4]	15	1986	AUB; abdominal mass	Abdominal myomectomy	16
Horejsi et al. [8]	15	1988	Not Informed	Abdominal Hysterectomy + BSO	Not Informed
Heimer et al. [14]	15	1991	Abdominal pain; fatigue	Abdominal myomectomy	17
Morad et al. [10]	15	1993	AUB; abdominal mass	Abdominal myomectomy	7
Fields et al. [6]	16	1996	22 weeks pregnant; periumbilical pain	Expectation	Not informed
Nguyen Duc et al. [18]	15	2003	AUB; abdominal mass; anemia	Not informed	Not informed
Bekker et al. [11]	15	2004	AUB; abdominal pain	Abdominal myomectomy	26
Grapsa et al. [13]	16	2006	AUB; abdominal mass	Abdominal myomectomy	30
Diesen et al. [12]	14	2008	Dysmenorrhea; abdominal distention	Abdominal myomectomy	15
Perkins et al. [17]	17	2009	Pelvic mass	Abdominal myomectomy	10
Karim et al. [19]	16	2010	Pelvic mass; increased abdominal volume	Abdominal myomectomy	25
Khorrami et al. [7]	17	2011	AUB; refractory anemia	Hysteroscopic myomectomy	3
Taskin et al. [5]	16	2011	Pelvic mass; mass protruding through vaginal opening	Hysteroscopic myomectomy	4
Wright et al. [9]	14	2011	AUB; abdominal pain; increased abdominal volume	Abdominal myomectomy	12
Naiditch et al. [15]	15	2011	Pelvic mass; abdominal pain; associated ovarian teratoma	Abdominal myomectomy and teratoma resection	6
Perez-Colon et al. [16]	15	2011	AUB	Not Informed	5
Kayadibi et al. [20]	15	2014	Pelvic mass; increased abdominal volume	Abdominal myomectomy	20

AUB – abnormal uterine bleeding; BSO – bilateral salpingoophorectomy.

Since then, some reports were published on fibroids in this specific population. In 1981, Augensen *et al.* described the case of a 15 year old patient that presented with abnormal uterine bleeding, urinary retention and an abdominal mass [3]. She underwent an abdominal myomectomy, with the intraoperative finding of a 10 cm leiomyoma, and was free of disease after one year of follow-up. In 1986, a new case was published by De Rooy *et al.*, describing another 15 year old adolescent who presented with hemorrhagic shock two days after the diagnosis of a pelvic mass suspicious for leiomyoma. Abdominal myomectomy was the offered treatment after an emergency laparotomy, although the intraoperative findings suggested a malignant tumor. Histopathology of the lesion diagnosed leiomyoma and there was no recurrence after 5 years of follow-up [4].

Among identified cases, average age was 15.35 years (14–17 years), mean diameter was 12.28 cm (3–30 cm) and median diameter was 10 cm. Most patients presented with symptoms (87.5%), the most frequent ones being increased uterine bleeding in frequency and/or intensity (10/18), abdominal pain (6/18) and perception of abdominal mass or increased abdominal volume (8/18). One of the patients had, as initial presentation, the exteriorization of a mass through the vaginal introitus, without any other complaints [5]. Another patient was pregnant when diagnosed, and was complaining only of periumbilical pain [6]. Three patients had symptoms related to anemia, with two them eventually needing transfusion. One patient presented with urinary retention due to compression by the enlarged fibroid [3].

Almost all patients were managed through myomectomy (88.2%), with two of them performed hysteroscopically [5,7]. Horejsi *et al.* reported on the case of a 15 year old patient who underwent a hysterectomy with bilateral salpingoophorectomy in 1988. A radical procedure was indicated due to the extension of the lesion and the presence of severe adhesions, according to the reporting authors, with no further details [8]. Wright *et al.* described the case of a 14 year old adolescent with abnormal uterine bleeding, pain and increased abdominal volume, in which a large fibroid (16 cm in diameter) was removed through an abdominal myomectomy and the patient was given a continuous progestagen postoperatively. After 6 months, there was recurrence of the disease, with an 11 cm tumor identified. A new myomectomy was performed, this time robotically, after which another recurrence was observed, with a smaller 3 cm tumor. Expectant management was offered and the patient followed-up for one year with no alterations [9]. Another report, by Morad *et al.,* described a 15 year old adolescent, who underwent abdominal myomectomy for a 7-cm tumor and became pregnant after 8 months of follow-up. There were no adverse events during pregnancy or delivery, which was carried out by cesarean [10]. The patient that was already pregnant at diagnosis was expectantly managed, with no intervention. Pregnancy outcome, however, was not reported [6]. In one of the hysteroscopic myomectomies reported, vaginal resection of a partly exteriorized leiomyoma was performed before completing the hysteroscopic resection [5].

Average follow-up was 1 year and 8 months (3 months to 6 years), and only one recurrence was reported, which occurred after 6 months after treatment. None of the cases were managed with medical treatments and, apart from the two hysteroscopic resections, no other minimally invasive non-surgical techniques (uterine artery embolization, magnetic resonance guided focused ultrasound) was employed.

## Discussion

The observation of symptomatic uterine fibroids among pediatric and adolescent population is an uncommon event. Thus, the characteristics of such disease in this specific group are not well known, and the most appropriate treatment is still not defined.

The etiology of uterine fibroids is generally unknown. It is known that they are monoclonal tumors, originating from a single cell that transformed and became neoplastic, and that almost half of them show chromosomal abnormalities [1]. In one of the reported cases, cytogenetic study was performed in the resected tumor, and a translocation between chromosomes 12 and 14 was identified [9]. Such translocation is one of the genetic aberrations commonly found in these tumors [21]. The precise factor that sets in motion a chain of events that lead to leiomyoma formation, however, is still unknown. Consequently, it is also unknown if the cases occurring in adolescents are related to some specific factor or not. It has been suggested that leiomyomas might originate from intrinsic anomalies in the myometrium; from congenitally elevated levels of sex steroids; and from endometrial injury acquired during menstruation [1]. Any of these theories can justify the appearance of such tumors in adolescents, after menarche, when endometrial sloughing and sex steroid exposition have already occurred, but they don’t explain the reasons why the lesions appeared sooner, rather than later in adulthood. The authors of one of the reports have theorized that the lesions were probably already present from an early age, or were congenitally acquired, and development occurred after menarche due to sex steroid stimulation [4], which is a plausible hypothesis, but one that lacks any evidence. The few reported cases contribute to this lack of knowledge.

Clinical presentation of uterine leiomyomas varies in any age, depending on individual characteristics of the tumors and the women affected. It is estimated that about 50% of uterine fibroids are asymptomatic, which is probably an understatement due to the great number of undiagnosed cases [22]. Among symptomatic women, the most frequently observed complaints are irregular or intense uterine bleeding, pelvic pain and symptoms related to compression by the tumors, such as urinary frequency or urgency. The presentation of such lesions among adolescents doesn’t seem to differ from the general population of women. Most reported cases showed symptomatic adolescents (87.5%). The number of reports, however, is small and the possibility of a significant number of undiagnosed cases or of expectantly managed asymptomatic cases among adolescents must be taken into account. An estimate may be drawn from an epidemiologic survey involving 21,479 women in 8 countries, which were evaluated through an online questionnaire. The study included 2,180 adolescents aged 15 to 19 years, among whom the estimated prevalence of leiomyoma based on self-reported diagnosis was 0.4% [23]. Such prevalence, despite being low, contrasts with the minimal number of symptomatic cases reported. It is possible that most teenagers who answered the survey don’t experience disturbing symptoms that justify seeking medical care.

Differential diagnosis of these tumors in adolescents is important. An increase in abdominal volume due to a pelvic mass in this age group must always alert the attending physician to the possibility of an adnexal tumor. In one of the reported cases, a large lesion, with 30 cm in diameter, was initially interpreted as an ovarian neoplasm and only during laparotomy the diagnosis of uterine leiomyoma was made and a myomectomy carried out [13]. Other tumors originating from the body of the uterus might also affect teenagers and must be remembered. Müllerian adenosarcomas are low grade tumors, most commonly observed in elderly women, but a series of nine cases affecting adolescents was reported by Andrade *et al.*, who described uterine bleeding and the exteriorization of a tumor through the vaginal introitus as the main presenting findings [24]. Cases in which a leiomyoma protrudes through the cervix in adolescents must also be differentiated from Sarcoma Botryoides, a rare tumor originating from the vaginal mucosa, which affects mainly children and adolescents, and presents through the appearance of a mass in the vaginal opening. It is the most common vaginal neoplasia in girls up to 10 years-old [5].

Regarding the treatment of leiomyomas occurring during adolescence, experience is limited to the published cases, and there are no guidelines that shed light specifically on this situation. Amidst the reported cases, all patients that were treated had their leiomyomas resected with uterine preservation, with the objective of preserving fertility, except for one patient, in which a radical surgery was performed, but the reason for such treatment were not clear. However, surgical treatment has not been the only option to offer symptomatic women. Medical treatments and minimally invasive techniques are available and are routinely used in reproductive aged women. The use of such treatments in adolescents, however, lacks any evidence, and little is known about their applicability in this group of patients.

Since leiomyomas are such an uncommon finding in this young population, the treatment offered to the few symptomatic cases encountered was extrapolated from that offered to older reproductive aged women. Myomectomy, although invasive, is probably the most adequate treatment, since it is durable, owing to the low recurrence rates of fibroids, preserves fertility, and doesn’t interfere with the hormonal milieu of the developing teenager. As for the real incidence of such disease in this population, although 85% of the treated adolescents of the reports presented with symptoms, it must be remembered that they represent a selected population, which sought medical care due to their complaints. An epidemiological survey estimates a prevalence of 0.4% of such disease among teenagers. Consequently, as it occurs in older women, most adolescents with leiomyomas are probably asymptomatic.

Newer treatments for uterine fibroids have been developed, but we don’t know if they will be efficient for adolescents. Ulipristal is a selective progesterone receptor modulator (SPRM) have been successful to reach amenorrhea in 70-80% of women, with a faster median onset of 4 days to reach it and less adverse effects when compared to GnRH analogues [25]; however, this drug was not tested in women under 18 years. Aromatase inhibitors are similar to GnRH analogs; however, they were not tested in a younger population in trials that were performed [26]. Other minimally techniques, such as uterine artery embolization (UAE) and high-intensity focused ultrasound (HIFU), have shown high rates of efficacy in women over 18 years, with no case series in women under 18 years with abnormal bleeding secondary to fibroids; only one case was reported about UAE in a 12-year-old girl with an hematological disorder and life threatening heavy menstrual bleeding successfully treated [27].

## Conclusion

Certainly, it is noted that leiomyomas’ biologic behavior in adolescents seems to be different from that of older women, but their molecular characteristics still haven’t been analyzed. Biological studies should be performed in order to determine whether are differences among its pathophysiology. Optimal treatment is still not defined, but myomectomy has several advantages in this population, considering their possible fertility plans. And last but not least, leiomyomas must be remembered as an important differential diagnosis of pelvic mass in adolescents.
